# Targeting α_ν_β_3 _and α_ν_β_5 _inhibits photon-induced hypermigration of malignant glioma cells

**DOI:** 10.1186/1748-717X-6-132

**Published:** 2011-10-06

**Authors:** Stefan Rieken, Daniel Habermehl, Angela Mohr, Lena Wuerth, Katja Lindel, Klaus Weber, Jürgen Debus, Stephanie E Combs

**Affiliations:** 1 University Hospital of Heidelberg, Department of Radiation Oncology, Im Neuenheimer Feld 400, 69120 Heidelberg, Germany

**Keywords:** glioma, radiotherapy, migration, integrin, vitronectin

## Abstract

**Background:**

Sublethal photon irradiation was recently suspected to increase tumor cell motility and promote locoregional recurrence of disease. This study was set up to describe mechanisms underlying increased glioma cell migration through photon irradiation and to analyse the modifiability of photon-altered glioma cell motility by integrin inhibition.

**Methods:**

Eight μm pore size membranes were coated with vitronectin (VN), collagen I and collagen IV. U87 and Ln229 glioma cells were analysed in migration experiments with and without radiotherapy (RT), serum stimulation and addition of monoclonal antibodies directed to human integrins α_ν_β_3 _and α_ν_β_5_. Quantitative FACS analysis of integrins was performed in U87 and Ln229 glioma cells following RT. Statistical analysis was performed using Student's *t-*test.

**Results:**

Glioma cell migration is serum-dependent and can be increased by photon RT which leads to enhanced expression of Vn receptor integrins. Blocking of either α_ν_β_3 _or α_ν_β_5 _integrins by antibodies inhibits Vn-based migration of both untreated and photon-irradiated glioma cells.

**Conclusions:**

Peripheral glioma cells are at risk of attraction into the adjacent healthy brain by serum components leaking through the blood brain barrier (BBB). Radiation therapy is associated with upregulation of Vn receptor integrins and enhanced glioma cell migration at sublethal doses. This effect can be inhibited by specific integrin blockade. Future therapeutical benefit may be derived from pharmacological integrin inhibition in combination with photon irradiation.

## Introduction

Despite continuously evolving therapy regimes including extensive neurosurgery, multiagent chemotherapy, and dose-escalated conformal radiotherapy, primary brain tumors have not ceased to account for high lethality after short periods of time in most patients. Deep locoregional tumor cell infiltration that eludes modern imaging techniques and hampers complete local resection was accounted responsible for early relapse and spread of disease throughout the brain. Current glioma therapy involves surgical tumor resection followed by adjuvant radiotherapy combined with concomitant and adjuvant chemotherapy [1].

As opposed to the tissue they originate from, most tumor cells, including malignant glioma cells, possess the unique ability to migrate and adhere to various surfaces, displaying polyligand-induced motile phenotypes where non-malignant cells are subjected to strictly regulated tissue architecture. Deregulated tumor cell migration is typically associated with infiltration and dissemination, resulting in local disease progression and metastases, both of which account for the majority of cancer-associated deaths. In gliomas abundant promigratory mediators have been identified including lipids and peptides, all of which can be detected in serum reaching the brain via the tumor-disrupted BBB [2-6].

Besides factors of the microenvironment surrounding the tumor, also its treatment may effect the migratory behavior of tumor cells. Radiation therapy, which is implemented in virtually all concepts of glioma treatment, was recently observed to increase tumor cell motility *in vitro *at sublethal doses < 3 Gray (Gy) [7,8]. Increasing cellular movement in malignant gliomas would undermine the therapeutical intent and possibly impose a greater risk of deep locoregional tumor infiltration and metastasization *in vivo *onto the patients than even without therapy. Furthermore, photon irradiation is kown to modulate the expression of extracellular matrix proteins and thus alter the motility-determining environment of malignant gliomas [9].

Depending on their tissue of origin, tumor cells employ a variety of ECM proteins to adhere to and migrate on. Primary brain tumors are known to produce and contain abundant amounts of collagens and other ECM components that promote increased motility, induce invasion and clinically account for poor local control [10,11]. Molecular therapies have long been introduced into the treatment of malignant gliomas and have defined epithelial and vascular growth factor but also integrin receptors as promising targets [12-14]. Integrin signalling is known to significantly impact glioma cell motility but also survival, and has therefore emerged as a promising approach to targeted glioma treatment [15]. To date, only little data exists addressing the impact that a combination of photon irradiation and integrin-inhibition may have on glioma cell migration. This study was set up in order to characterize ECM-based motility of U87 and Ln229 glioma cells after photon irradiation and to analyse the impact of inhibition of Vn receptor integrins in combination with radiotherapy.

## Materials and methods

### Cell culture

U87-MG glioma cells were purchased from LGC Promochem (ATTC HTB-14), and kept at 37°C and 5% CO_2 _in DMEM (FG0415 Biochrom AG) supplemented with 1% Penicilline/Streptomycine and 10% FCS. Ln229 glioma cells were purchased from LGC Promochem (ATTC CRL-2611), and kept at 37°C and 5% CO_2 _in DMEM (FG0415 Biochrom AG) supplemented with 1% Penicillin/Streptomycin and 10% FCS. Twenty-four hours before adhesion and migration experiments, cells were serum starved in DMEM containing 1% Penicilline/Streptomycine and 0.5% FCS. Passaging of cells was performed every week.

### Surface coating with extracellular matrix proteins

For migration assays, polycarbonate membranes with 8 μm pores were coated with 50 ng/cm^2 ^vitronectin, 0.5 μg/cm^2 ^collagen I and 0.5 μg/cm^2 ^collagen IV over night at 4°C and washed in twice destillated and salt-free water prior to the experiments.

### Migration assay

Five × 10^3 ^cells were loaded in the upper chamber of a 48-well modified microchemotaxis chamber (Multiwell Chemotaxis Chamber, Neuro Probe). The lower well contained cell culture medium with 0.5% FCS and chemoattractants as indicated. Lower and upper chambers were separated by a 8 μm pore size polycarbonate membrane, that had been coated with vitronectin (50 ng/cm^2^), collagen I (0.5 μg/cm^2^) and collagen IV (0.5 μg/cm^2^) 24 hours before the start of migration. Cells were serum-starved in medium containing 0.5% FCS 24 hours prior to the start of migration. Radiation treatments were performed 24 hours before assessment of migration. Before staining and mounting of Boyden chamber membranes, non-migrated cells on the upper filter side were removed by drawing the filter over a wiper blade at least twice. Cytoseal XYL mounting medium was used for filter preservation (Richard-Allan Scientific). Transmigrated cells were stained with DiffQuick^® ^and counted with a Leica DC300F microscope. Integrin blockade was performed using monoclonal antibodies directed against α_ν_β_3_- (MAB3050, R&D) and α_ν_β_5_-integrins (MAB 2528, R&D). All assays were done in at least triplicates and wells were counted by an investigator blinded to experimental set-up. Cell numbers are expressed as multiples of controls or as proportion of inputs.

### FACS analysis

Twenty-four hours after irradiation, cells were fixed with 70% ethanol and stained with a PE-labelled anibody directed against α_ν_β_3 _(555505, BD) and a FITC-labelled antibody directed against α_ν_β_5 _(FAB2528P, R&D). Ln229 and U87 glioma cells were analysed with a three-colour FACScan flow cytometer and CellQuestPro software (BD Biosciences). Results are displayed with histogram plots and subsequent quantitative analyses.

### Radiation treatment

Photon radiation was performed using with a 6 MeV linear accelerator (Siemens, Erlangen, Germany). We applied single doses of 2, 5, and 10 Gy 24 hours prior to migration experiments.

### Statistics

All migration experiments and FACS analyses were performed at least three times. Modified Boyden Chamber assays were set up in triplicates and analysed by an investigator blinded to experimental setup. Data are displayed as means ± standard deviation (SD). Comparisons between two groups were performed with Student's *t*-test.

## Results

### Glioma cell migration is promoted by serum exposition

**Figure 1:**
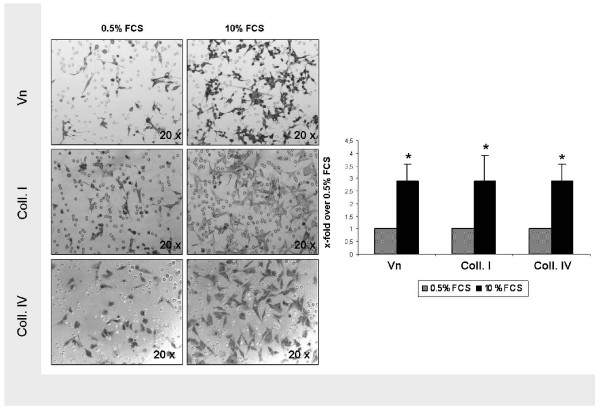
**FCS stimulation of U87 glioma cell migration**. Transmigration through DiffQuik^®^-stained 8 μm pore size polycarbonate membranes coated with Vn, collagen I and IV with and without serum stimulation (magnification, x20). Induction of transmigration by a 10% FCS gradient displaying a 2.91-fold increase on Vn (p < 0.0001), a 2.90-fold increase on collagen I (p = 0.0004), and a 2.89-fold increase on collagen IV (p < 0.0001). Display of mean value ± standard deviation (SD); statistical analysis using Student's *t*-test (*, indicating significance p < 0.05)

Modified Boyden chamber assays were performed to analyse transmigration of U87 glioma cells through 8 μm pore size polycarbonate membranes coated with Vn and collagen I and IV. In order to mimic a disturbed BBB with leakage of blood-borne mediators, such as often detected in brain tumors, cells were attracted by serum. This gradient clearly increased the number of transmigrated glioma cells (Figure 1). Compared to unstimulated conditions, serum exposition using a 10% FCS gradient increased chemotactic transmigration by a factor of 2.91 (p < 0.0001), 2.90 (p = 0.0004) and 2.89 (p < 0.0001) through Vn-, collagen I- and collagen IV-coated filters (Figure 1).

### Sublethal photon irradiation enhances glioma cell migration

**Figure 2:**
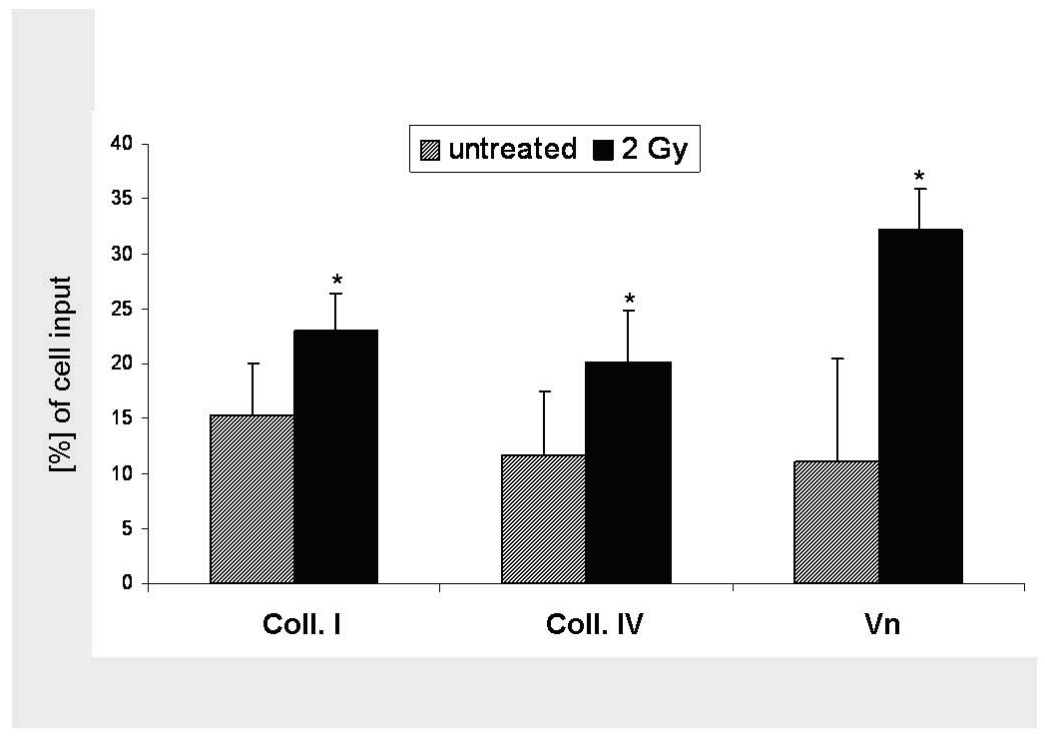
**Photon stimulation of U87 glioma cell migration**. Graphical analysis of induction of transmigration by a single photon doses of 2 Gy through membranes coated with collagen I (15.3% vs. 22.9% [p = 0.0002]), collagen IV (11.6% vs. 20.1% [p = 0.01]) and Vn (11.1% vs. 32.3% [p < 0.0001]). Display of mean value ± standard deviation (SD); statistical analysis using Student's *t*-test (*, indicating significance p < 0.05)

Since photon irradiation is implemented in most glioma treatment protocols, we irradiated U87 glioma cells with single photon doses of 2 Gy and analysed transmigration 24 hours afterwards. Single photon doses of 2 Gy promoted glioma cell transmigration (Figure 2). On collagen I-coated surfaces, the fraction of transmigrated cells was increased from 15.3% to 22.9% (p = 0.0002); on collagen IV transmigration was raised from 11.6% to 20.1% (p = 0.01). The highest photon stimulation of migration was detected on Vn-coated membranes, where an increase from 11.1% to 32.3.% was observed (p < 0.0001).

### Photon irradiation increases expression of vitronectin receptor integrins α_ν_β_3 _and α_ν_β_5_

**Figure 3:**
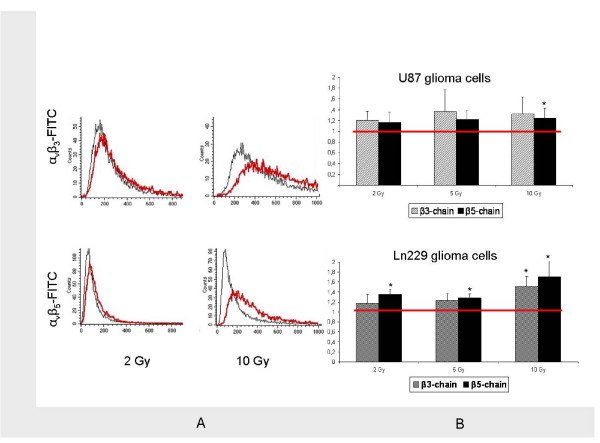
**Photon-induced stimulation of integrin expression**. A: FACS analysis of α_ν_β_3 _(upper row) and α_ν_β_5 _(lower row) expression with (red) and without (black) photon irradiation with single doses of 2 Gy (left) and 10 Gy (right). B: Graphical analysis of integrin α_ν_β_3 _(gray bars) and α_ν_β_5 _(black bars) expression with (bars) and without (red line) irradiation with single photon doses of 2, 5, and 10 Gy in U87 (upper chart) and Ln229 (lower chart) glioma cells. Display of mean value ± standard deviation (SD); statistical analysis using Student's *t*-test (*, indicating significance p < 0.05)

Quantitative FACS analyses using PE- and FITC-labelled antibodies directed against the Vn receptor integrins α_ν_β_3 _and α_ν_β_5 _on cell surfaces were performed in order to investigate the effects of photon RT on integrin expression. We found that single doses of 2 and 10 Gy caused a rightshift in the FACS histograms (Figure 3A) and increased the expression of both α_ν_β_3 _and α_ν_β_5 _24 hours after irradiation (Figure 3B). This rightshift was consistent for all doses tested and for both α_ν_β_3 _and α_ν_β_5 _in U87 cells. However, statistical significance was only reached for expression of α_ν_β_5 _after 10 Gy (p < 0.05).

To confirm that this phenomenon was not limited to U87 glioma cells (Figure 3B, upper row), we analysed Ln229 glioma cells and found the same phenotype of enhanced integrin expression following photon RT (Figure 3B, lower row). Photon-induced upregulation of integrins was particularly strong for α_ν_β_5 _in Ln229 cells, where statistical significance was reached for all photon doses tested (p < 0.05).

### Inhibition of α_ν_β_3 _and α_ν_β_5 _significantly impairs photon-induced hypermigration

**Figure 4:**
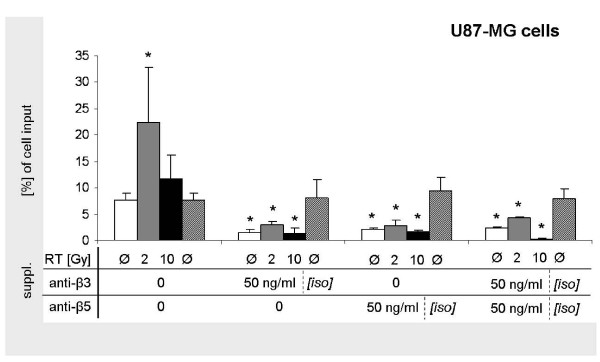
**Inhibiton of U87 glioma cell migration by integrin inhibition**. Quantitative analysis of Vn-based transmigration of U87 glioma cells following single photon doses of 0, 2, and 10 Gy without and with the addition of 50 ng/ml anti-α_ν_β_3_- and -α_ν_β_5_-antibodies and corresponding isotype controls.

**Figure 5:**
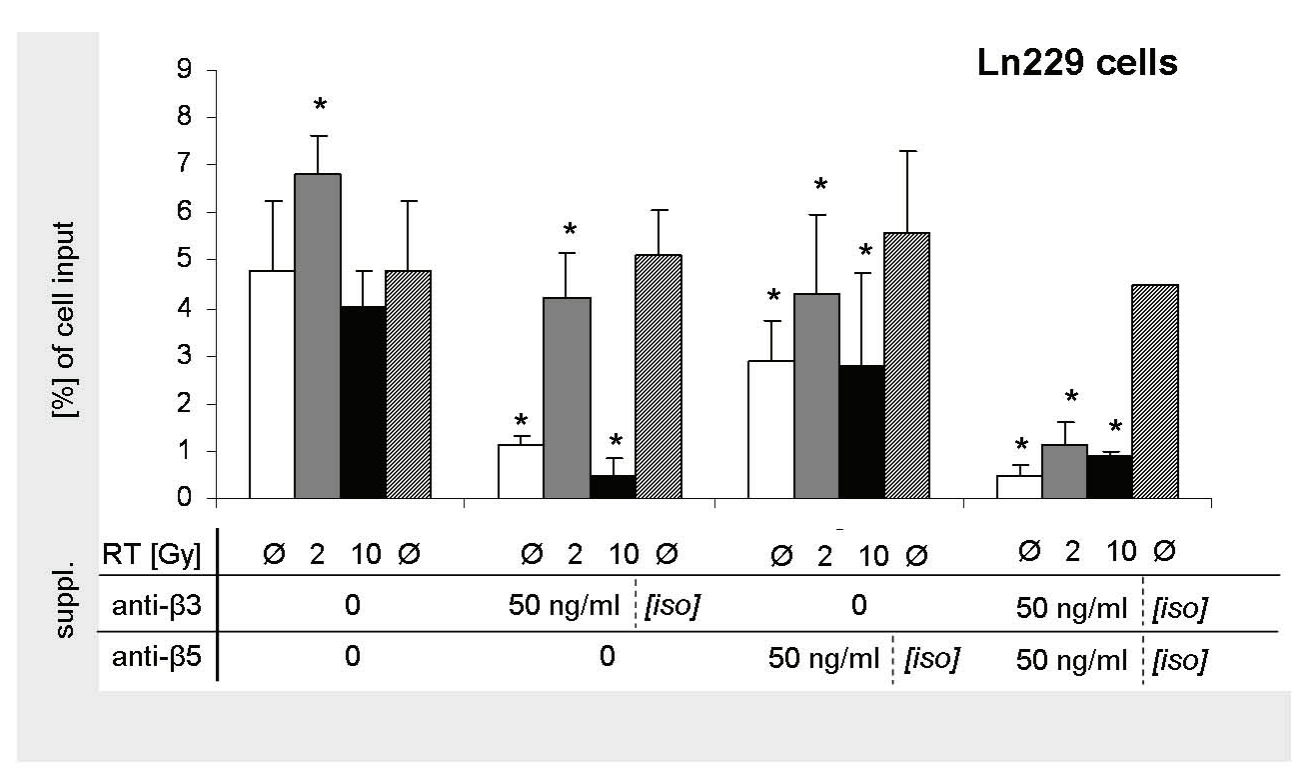
**Inhibiton of Ln229 glioma cell migration by integrin inhibition**. Quantitative analysis of Vn-based transmigration of Ln229 glioma cells following single photon doses of 0, 2, and 10 Gy without and with the addition of 50 ng/ml anti-α_ν_β_3_- and -α_ν_β_5_-antibodies and corresponding isotype controls. Display of mean value ± standard deviation (SD); statistical analysis using Student's *t*-test (*, indicating significance p < 0.05)

In order to analyse the effect of the integrin-disruption, we used Vn receptor-targeting anti-α_ν_β_3_- and -α_ν_β_5_-antibodies in Vn-based migration experiments and found that concentrations of 50 ng/ml impaired glioma cell migration. Both single inhibition of either α_ν_β_3 _and α_ν_β_5 _caused significantly reduced transmigration in both U87 (Figure 4) and Ln229 (Figure 5) cells. Besides in untreated cells, also photon-stimulated cells were significantly inhibited from migrating through Vn-coated membranes. Strongest inhibition was achieved when both anti-α_ν_β_3_- and anti-α_ν_β_5_-antibodies were added. Addition of isotype controls did not affect migration.

## Discussion

In the present manuscript we investigated a repeatedly described phenomenon of photon-induced tumor cell migration and found that promotion of glioma cell chemotaxis by photon doses of 2 Gy was particularly strong on Vn-coated surfaces. Providing a molecular explanation for this phenotype, we detected a consistent trend of increased expression of the Vn receptor integrins α_ν_β_5 _and α_ν_β_3 _following photon RT. Pharmacological disruption of these integrins reversed the phenomenon of photon-triggered migration and may therefore serve as novel approach for combination treatments.

Tumor cell exposition to serum components at the disturbed BBB in glioma patients is generally accepted to account for locoregional infiltration and early tumor relapse. Hartman *et al *have shown that in 75% of all recurrent malignant glioma, relapse occurs within the initial edema conformation [3]. Tumor-associated brain edema represents breakdown of the BBB and allows the entry of chemotactic serum compounds into the widened interstitial spaces thus facilitating cell motility and promoting clinical tumor infiltration [16,17]. We supplemented cell culture medium with 10% FCS, thus, exposing the cells to a physiological combination of serum compounds and found that migration was significantly enhanced on Vn- and collagen-coated surfaces. Our findings support previously established theories of tumor cell dissemination along serum gradients and relapses of disease occurring in environments with a promigratory milieu. Many models were established in order to explain the deregulation of tumor cell motility, including tumor cell specific adhesion, migration, and infiltration. In malignant gliomas, disturbance of the BBB allows exposure of peripheral tumor cells to serum components which the healthy brain tissue is physiologically separated from [18]. Independently of prior treatments, the site of exposure to serum in the tumor periphery is known to represent the typical location for tumor recurrence [3]. Various authors have found glioma cells to migrate towards serum proteins, such as albumin [2], and phospholipids, such as lysophosphatic acid [4]. Also, blood-bourne chemokines such as CXCL-12 are known to attract tumor cells and contribute to their wide spread within the brain [5]. Due to continuous ligand exposition, peripheral glioma cells are at highest risk of chemotactic distraction.

Photon irradiation has a substantial role in modern interdisciplinary cancer therapies. It is implemented in most glioma treatment regimes and improves local control rates and survival [19]. However, several reports have described sublethal photon doses lower than 3 Gy to trigger tumor cell migration [7,8,20]. These doses are commonly applied in fractionated RT and may therefore impose a risk of enhanced tumor cell invasion and dissemination onto the patients. Wick *et al *showed that single photon doses as high as 6 Gy increased chemotactic glioma cell migration along with upregulated integrin expression and enhanced activity of matrix metalloproteinases [8]. Knowing that peripheral tumor cells are at highest risk of ligand-induced distraction and may at the same time be triggered to migrate by sublethal photon RT requires either conceptual changes in RT, such as wider safety margins or use of particle irradiation or administration of additional motility-suppressive agents.

We demonstrated that glioma cell migration was significantly enhanced by serum exposition on collagen I and collagen IV. Collagen I is detected abundantly in the outer lining of the brain parenchyma, the glia limitans externa. Collagen IV is typically found in the basement membrane of blood vessels and critically contributes to pathological vessel formation in malignant disease. Both are known to represent preferred substrates for invasive glioma cells which have been shown to migrate along distinct anatomical interfaces [21-23]. In our experiments, we detected robust - both serum- and photon-responsive - glioma cell interaction with both collagen I and IV. However, due to the large family of collagen-binding integrins and their presence in multitudinous tissues essentially contributing to organ architecture, targeting collagen-cell-interaction does not appear to be safely feasible [24].

Vn and its main binding integrins, α_ν_β_3 _and α_ν_β_5_, are found in high density at the infiltrating invasion front of high grade gliomas [25,26] and inhibition of α_ν_β_3 _was demonstrated to slow down glioma cell migration [27]. Furthermore, soluble Vn was identified as one of the major factors in serum and cerebrospinal fluid to induce glioma cell migration [28]. Depletion of Vn in these fluids caused significant decrease in migration. Vn therefore contributes to the malignant phenotype of gliomas and also affects their sensitivity to treatment. The integrins α_ν_β_3 _and α_ν_β_5 _represent physiological Vn receptors, and they were described to significantly influence the radiosensitivity of gliomas. Their downstream signalling involves integrin-linked kinases and GTPases, and their ligand-induction resulted in enhanced radioresistance *in vitro *[29]. Pharmacological disruption of these signalling cascades would, therefore, be an auspicious strategy in glioma treatments and permit additional radiosensitization. Our results support the hypothesis of Vn as a radioprotective ECM protein, because the highest photon-induced migration was detected on Vn-covered membranes, where sublethal RT yielded an almost twofold increase.

We performed flow cytometry analyses and found a trend toward increased expression of α_ν_β_3 _and α_ν_β_5 _following photon RT, while expression of β_1_-integrins remained stable (data not shown). Our results are in line with previously published results data on photon-induced integrin upregulation in endothelial cells [30], lung cancer cells [31], colon carcinoma cells and also glioma cells [20]. At low photon doses, integrin expression corresponds to the significantly increased migration of glioma cells on Vn-coated membranes following RT, whereas increased integrin expression following 10 Gy was associated with unaltered migration. This indicates that at higher photon doses, further mechanism must influence tumor cell motility. We confirm data previously published by Goetze et al., who concluded that radiotherapy may affect tumor cell migration in partially opposing ways [20].

Since integrin signalling is susceptible to pharmacological disruption, we added Vn receptor antibodies in order to analyse the modifiability of glioma cell migration. The addition of either anti-α_ν_β_3_- and -α_ν_β_5_-antibodies reduced transmigration of both untreated and irradiated U87 and Ln229 cells. Blocking α_ν_β_3 _yielded slightly stronger inhibition than did sole blocking of α_ν_β_5_. This is in line with several previous works that have identified α_ν_β_3 _to be both predominant in Vn-associated migration and also mediate signalling effects of further ECM components, such as fibronectin [26,32-34]. However, the effect of integrin inhibition could still be enhanced when anti-α_ν_β_3_- and -α_ν_β_5_-antibodies were combined, thus fully abrogating Vn signalling effects. Our experiments show that a photon-derived induction of α_ν_β_3 _and α_ν_β_5 _expression with consecutively increased Vn-based glioma cell migration at sublethal doses, can be successfully counteracted by combining RT with targeted therapies.

## Conclusion

Photon RT with single doses of 2 Gy increases glioma cell migration via integrin-induction and may, therefore, enhance the risk of tumor cell spread and infiltration. Integrin-targeting antibodies effectively antagonize this photon-induced increased migration. Therefore, they represent a novel and promising approach to combination treatments with fractionated photon RT especially during early fractions when lethal doses have not been reached, yet. Higher photon doses do not promote cell migration despite integrin upregulation, and must therefore be interpreted with caution. Further studies are needed to evaluate the clinical impact of these *in vitro *findings in a clinical setting.

## List of abbreviations

RT: radiotherapy; FCS: fetal calf serum; Vn: vitronectin; ECM: extracellular matrix; BBB: blood brain barrier; Gy: Gray

## Competing interests

There are no conflicts of interest to declare. Stefan Rieken was supported by the medical faculty of Heidelberg (PostDoc grant).

## Authors' contributions

SR conceived of the study design, performed all experiments and wrote the manuscript. DH and AM helped to analyse migration experiments. LW was responsible for irradiation of the cells and for FACS analysis of integrin expression. KL and KW supervised irradiation experiments. JD contributed with regard to content, scientific context and financial support. SC conceived of the study and helped to write and finalize the manuscript. All authors helped with the interpretation of the data, read and approved the final manuscript.
